# Immune Checkpoint Inhibitors in Merkel Cell Carcinoma of the Skin: A 2025 Comprehensive Review

**DOI:** 10.3390/cancers17193272

**Published:** 2025-10-09

**Authors:** Patricia Tai, Omar Alqaisi, Suhair Al-Ghabeesh, Lorent Sijarina, Edward Yu, Aoife Jones Thachuthara, Avi Assouline, Osama Souied, Kimberly Hagel, Kurian Joseph

**Affiliations:** 1 Oncology Department, University of Saskatchewan, Saskatoon, SK S7N 5A2, Canada; 2 Nursing Department, Al-Zaytoonah University, Amman P.O. Box 130, Jordan; omaralqaisi119@gmail.com (O.A.);; 3 Faculty of Medicine, University of Prishtina, 10000 Prishtina, Kosovo; lorent.sijarina@student.uni-pr.edu; 4 Oncology Department, Western University, London, ON N6A 3K7, Canada; 5 Medical Oncology Department, Cork University, T12 K8AF Cork, Ireland; 6 Oncology Department, Collège de Médecine des Hôpitaux de Paris, Cedex 12, 75 610 Paris, France; 7 Oncology Department, University of Alberta, Edmonton, AB T6G 2R3, Canada

**Keywords:** Merkel cell carcinoma, skin cancer, immunotherapy, immune checkpoint inhibitor, PRISMA, systemic therapy, review, recommendations, clinical trials

## Abstract

**Simple Summary:**

Merkel cell carcinoma is a rare and aggressive form of skin cancer. While immunotherapy has transformed its management, published data remain limited. Therefore, we conducted this review to evaluate the current landscape, with the ultimate goal of improving patient care. Merkel cell carcinoma is known to spread to lymph nodes and distant organs. We searched four databases for publications on immune checkpoint inhibitors, which may be administered either before or after surgery. In cases of unresectable or advanced disease, they can be used as a standalone treatment. Our summarized findings highlight the need for further clinical research to guide future therapeutic approaches.

**Abstract:**

**Objective:** Merkel cell carcinoma (MCC) is a rare and aggressive form of skin cancer. Although immunotherapy has transformed MCC management, published data remain limited. This comprehensive review evaluates current evidence on immune checkpoint inhibitors (ICIs) in MCC, in relation to other treatment modalities such as surgery and radiotherapy. **Methods:** Peer-reviewed articles published between January 2000 and August 2025 were searched manually in four databases: Scopus, ScienceDirect, PubMed and MEDLINE, using the keywords “Merkel cell carcinoma” AND “immunotherapy” AND “immune checkpoint inhibitors”. The Preferred Reporting Items for Systematic reviews and Meta-Analyses (PRISMA) methodology was employed. **Results:** ICIs can be given in different settings: (A) *Neoadjuvant*: The CheckMate 358 trial reported a 54.5% response rate among 33 radiologically evaluable patients treated with nivolumab, each showing over 30% tumor reduction. (B) *Adjuvant*: (1) The ADMEC-O phase II trial demonstrated improved disease-free survival with adjuvant nivolumab. (2) The ADAM phase III trial evaluates adjuvant avelumab in node-positive patients post-surgery/radiation, with common side effects including nausea, fatigue, and itching. (3) STAMP, a phase III trial, investigates pembrolizumab in stage I–III MCC. Both ADAM and STAMP have completed accrual and results are pending. (C) *Primary therapy*: KEYNOTE-017 and JAVELIN trials reported a 60% overall response rate and ~40% 3-year progression-free survival with first-line pembrolizumab or avelumab. Both agents also show promise as salvage therapies. **Conclusions:** ICIs demonstrate encouraging outcomes in MCC across various treatment stages. Continued research is essential to optimize treatment timing and integrate multimodal therapies.

## 1. Introduction

Merkel cell carcinoma (MCC) is a rare but highly aggressive neuroendocrine skin cancer, often associated with Merkel cell polyomavirus (MCPyV) or ultraviolet-induced mutations [1]. MCC is aggressive, with a mortality rate exceeding melanoma. Despite its low incidence, MCC carries a disproportionately high mortality rate, with five-year survival estimates ranging from 30 to 60% depending on stage and treatment modality. About 50% of patients develop lymph node metastases and 30% develop distant metastases. Locoregional recurrence occurs in up to 50% of cases.

Historically, treatment options were limited to surgery and radiation, with chemotherapy offering modest and short-lived benefits [1,2]. However, the emergence of immune checkpoint inhibitors (ICIs) has dramatically reshaped the therapeutic landscape.

Following the American Food and Drug Administration (FDA) approval in 2017, ICIs are now employed in both routine clinical practice and ongoing clinical trials [3,4]. The first ICI approved for MCC was avelumab (Bavencio), which received accelerated approval in March 2017. This milestone marked the first FDA-sanctioned treatment specifically for metastatic MCC [5,6].

Programmed Death-Ligand 1 (PD-L1) is a protein that plays a key role in regulating immune responses. PD-L1 binds to its receptor PD-1 on T cells, effectively putting the brakes on the immune system and allowing some cancer cells to evade detection [1,7]. ICIs, particularly agents targeting the PD-1/PD-L1 axis, has demonstrated durable responses in advanced MCC, prompting investigations into its role across the disease continuum—from neoadjuvant and adjuvant settings to primary and salvage therapy [1,8]. Yet, despite promising clinical outcomes, published data remain limited, and questions persist regarding optimal timing, integration with other treatment modalities, patient selection and mechanisms of resistance [1,9].

While other aggressive skin cancers, such as melanoma, have been extensively studied in the context of immunotherapy, MCC remains underrepresented in the literature despite its substantially higher disease-specific mortality and distinct biological profile [1,4]. Unlike melanoma, which is primarily driven by ultraviolet-induced mutations, MCC frequently arises in immunosuppressed or elderly patients and is often associated with viral oncogenesis through Merkel cell polyomavirus (MCPyV) [1]. These features may significantly influence tumor immunogenicity, treatment response, and resistance mechanisms [10]. Consequently, therapeutic strategies developed for melanoma or other skin cancers cannot be directly extrapolated to MCC. A focused review dedicated exclusively to ICIs in MCC is therefore essential to address the unique epidemiology, clinical challenges, and evolving therapeutic landscape of this rare malignancy.

This comprehensive review aims to synthesize current evidence and clinical practices scattered across the literature on ICIs in MCC, with a focus on key clinical trials, treatment strategies, and future directions. This work is unique for several reasons: (1) We followed the Preferred Reporting Items for Systematic reviews and Meta-Analyses (PRISMA) methodology, which, to our knowledge, has not previously been applied to MCC in the published literature. (2) As clinicians with direct experience managing this rare cancer, we provide expert recommendations to improve current treatment patterns. Our team compiled a robust database of 949 patients, including 303 who presented to our respective cancer centers in Canada, France, and Australia between March 1982 and February 2015 [11]. The remaining patients were drawn from individual patient data extracted from published case reports and series. (3) The most updated literature from 2025, along with major landmark studies, is summarized to provide practical and comprehensive bedside information for healthcare providers on MCC.

## 2. Methods

A comprehensive search was conducted across four databases: Scopus, ScienceDirect, PubMed, and MEDLINE to identify peer-reviewed studies on Merkel cell carcinoma (MCC) and immunotherapy published between January 2000 and August 2025. The search strategy utilized the keywords “Merkel cell carcinoma” and “immunotherapy” and “immune checkpoint inhibitors”. The review process adhered to the Preferred Reporting Items for Systematic Review and Meta-Analyses (PRISMA) guidelines for study identification, screening, and selection [12].

**Inclusion criteria** encompassed clinical trials, observational studies, systematic reviews, guidelines, meta-analyses and case reports that evaluate immunotherapy in MCC. **Exclusion criteria** were limited to articles not published in English and studies lacking immunotherapy-specific outcomes. The restriction to English-language publications were applied to ensure accurate data interpretation and avoid potential errors or bias introduced by translation. The most important studies are in English language anyway as we searched the four databases. Studies without specific immunotherapy outcome data were excluded to maintain focus on the relevant clinical endpoint.

**For data extraction,** two authors (O.A. and S.A.) independently reviewed each eligible study. They extracted key information, including the study design, patients’ population, immunotherapy regimen, response rates, progression-free survival (PFS), overall survival (OS), and any reported adverse events. **The extracted data** were then cross-checked between the two authors, and any discrepancies or disagreements were resolved through careful discussion and re-examination of the source of articles. A third researcher was available to arbitrate if consensus could not be reached; however, in practice, the two-authors reached agreement on all data points after discussion. This consensus approach ensured the reliability and accuracy of the extracted data. Finally, the included studies were categorized by treatment setting (neoadjuvant, adjuvant, or primary therapy) to facilitate subgroup analysis of the outcomes.

## 3. Results

**Figure 1:**
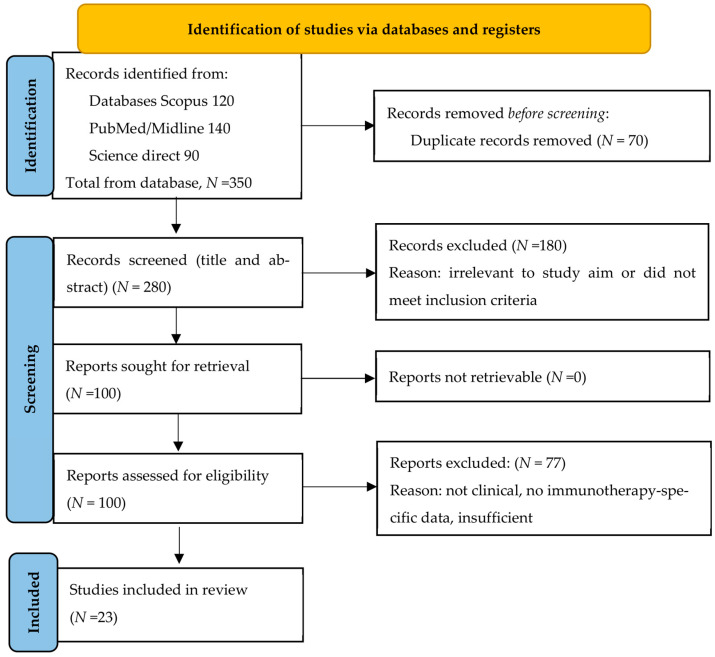
Flow diagram of comprehensive review for studies with the Preferred Reporting Items for Systematic reviews and Meta-Analyses (PRISMA) methodology.

The search and study selection process is summarized in the PRISMA flow diagram (Figure 1). A total of 350 records were identified from the databases: Scopus (*N* = 120), PubMed, MEDLINE (*N* = 140), and ScienceDirect (*N* = 90). After removing 70 duplicate entries, 280 records remained for screening. Following a title and abstract review, 180 records were excluded based on irrelevance to the study objectives or failure to meet inclusion criteria. Then the full texts of 100 articles were assessed for eligibility. Of these, some studies were excluded due to reasons such as lacking clinical data, insufficient information on immunotherapy intervention, or inappropriate study design. Finally, those articles which satisfied all requirements are summarized in this review (Table 1). We are hoping to capture most important retrospective and prospective studies, latest reviews and practice guidelines. It is impossible to include all MCC abstracts; otherwise, the manuscript would be too long. The basic principles are illustrated with the above information by our international team of experts on MCC (P.T., E.Y. and K.J.) in collaboration with experienced researchers from a few developing countries.

### 3.1. Critical Appraisal (Summary Table S1 in Supplementary Materials)

To strengthen the review, we conducted a rigorous assessment of trial quality and bias-risk using PRISMA guidelines and established tools. Overall, the evidence is limited by the scarcity of MCC studies, and most included trials have modest sample sizes and design constraints. Quality appraisal indicated that while several studies were well-designed (e.g., the ongoing ADAM trial is a randomized double-blinded placebo-controlled Phase III) [32], many others carried a moderate risk of bias. For instance, a recent meta-analysis noted that nine of 13 immunotherapy studies in MCC were rated “good” quality (NOS score ≥ 8) but the remainder were only “fair” (score 5–7) due to design limitations [9].

***Key methodological limitations identified across the trials include:***

- **Small Sample Sizes**—Most trials enrolled only a few dozen patients, reflecting MCC’s scarcity. Small sample size reduces statistical power and precision; for example, the neoadjuvant Checkmate 358 study had 33 patients [13] and KEYNOTE-017 enrolled 50, which constrains subgroup analyses and the detection of modest effects [33].
- **Lack of Randomized Controls trials**—Several pivotal studies were single-arm or non-randomized (e.g., CITN-09/KEYNOTE-017 and JAVELIN Merkel 200 [33,34]), relying on historical comparisons. The absence of a control arm increases potential selection bias and makes it difficult to attribute outcomes solely to the immunotherapy [13]. Ongoing randomized trials (ADMEC-O [18], ADAM [32], STAMP [35], I-MAT [36]) aim to address this, but their final results are pending [34].
- **Limited Blinding**—Among the randomized trials, blinding was inconsistently applied. Notably, the phase II ADMEC-O trial was open-label, meaning patients and investigators knew the assigned treatment [18]. Open-label designs can introduce performance and detection bias (e.g., differences in ancillary care or assessment of progression), especially for endpoints like radiographic DFS. By contrast, the ADAM trial’s double-blind design minimizes such bias [32].
- **Endpoint and Follow-up Constraints**—Many studies reported surrogate or short-term endpoints (objective response rate, 12 or 24 month progression/recurrence-free survival) with immature overall survival data. For example, the interim results of ADMEC-O showed a 9–10% improvement in 1–2-year DFS with adjuvant nivolumab, but this did not reach statistical significance and OS outcomes remain not yet mature [18]. Short follow-up can miss late recurrences and long-term toxicities [31].
- **Trial Execution and Reporting**—We noted variations in endpoint definitions and possible attrition biases. In ADMEC-O, an imbalance in post-surgery radiation between arms occurred, which could confound DFS results [34]. Additionally, the high dropout or non-evaluable rates in some trials were not always clearly reported, raising concern for attrition bias. Finally, publication bias is possible, as positive trials are more likely to be reported; our comprehensive search and PRISMA-based selection aimed to mitigate this, but the predominance of encouraging results suggests that small negative studies might be underrepresented [9].
- **Generalizability and Clinical Applicability** An expanded discussion is necessary to examine how well clinical trial findings translate to the broader MCC patient population. Because MCC is uncommon, clinical trials often involve highly selective patient cohorts, which may limit external validity. Key factors affecting generalizability include patient selection criteria, demographic representation, and real-world clinical context.
- **Patient Selection and Fitness:** Trial participants tended to be relatively fit (ECOG 0–1) with controlled comorbidities, whereas real-world MCC patients are often elderly with significant medical issues. The median age in trials (~65–70) is somewhat lower than the general MCC population, and frail patients were underrepresented [37]. Furthermore, strict eligibility criteria (organ function cut-offs, exclusion of recent malignancies, etc.) meant approximately one in five real-world oncology patients would not qualify for typical phase III trials [37]. Physicians also tend to enroll patients with better prognoses (younger, fewer comorbidities) [37]. This selection bias can inflate efficacy and reduce toxicity in trials compared to unselected populations. Clinicians must therefore judiciously extrapolate results to older or frailer patients, assessing whether the survival benefit outweighs risks in those less robust than trial subjects.
- **Immunosuppression and Viral Status**: A noteworthy proportion of MCC patients are immunosuppressed, for example, organ transplant recipients, human immunodeficiency virus (HIV)-positive, or with chronic lymphocytic leukemia, but such patients were largely excluded from trials [38]. Consequently, data on immunotherapy efficacy in immunosuppressed MCC are sparse. Emerging real-world evidence indicates these patients derive lower benefit—in a retrospective analysis, immunosuppressed MCC patients had an initial ICI response rate of ~50% versus ~62% in immunocompetent patients, along with higher progression and toxicity rates [38]. For example, 52% of immunosuppressed patients experienced serious immune-related side effects, compared to 37% of those with intact immunity. These findings suggest that while checkpoint inhibitors can be effective in immunosuppressed MCC, responses are attenuated and risks heightened, underscoring caution when generalizing trial outcomes to this subgroup. Additionally, the Merkel cell polyomavirus (MCPyV) status of patients might influence disease biology. Most trials enrolled both virus-positive and negative tumors without stratification, and encouragingly, responses have been observed in both groups for instance, pooled analyses show similar objective response rates regardless of MCPyV status [39]. However, since MCPyV prevalence differs by region (e.g., higher in North America/Europe, lower in Australia), the trial populations’ viral status mix may not reflect all settings. Ongoing research into virus-specific immunity may further clarify if virus-negative (UV-driven) MCC requires adapted immunotherapy strategies.
- **Geographic and Ethnic Representation:** The major immunotherapy trials in MCC have been conducted in North America, Europe, and Australia, with very limited participation from Asia, Africa, or South America [40]. Consequently, the ethnic diversity of trial cohorts was narrow—a large majority of patients were of White/European ancestry, mirroring MCC’s higher incidence in those populations. This homogeneity raises questions about applicability to other ethnic groups. While there is no evidence that tumor responses to PD-1/PD-L1 inhibitors differ by race, disparities in healthcare access and genetic backgrounds could influence real-world outcomes. The lack of diverse representation is an acknowledged challenge in oncology trials [9]. Efforts to broaden eligibility and include international sites are needed to ensure findings are globally relevant.
- **Real-World Clinical Settings:** Outcomes in routine practice may be less favorable than in trials. Outside the controlled trial environment, patients often have more advanced comorbidities, may receive treatment in non-specialized centers, or face interruptions in therapy. Indeed, studies across oncology had found that real-world survival can be significantly shorter than trial-reported survival—one analysis noted a ~6-month decrement in median overall survival for real-world patients on new cancer therapies [41]. For checkpoint inhibitors specifically, real-world patients appear to experience diminished benefit compared to trial patients in I-MAT trial [36], likely due to the inclusion of higher-risk individuals. In MCC, practical issues such as managing immune-related adverse events in older patients, or continuation of therapy beyond progression, may differ from protocol-driven trials. All these factors mean that the impressive 3-year survival ~60% seen with pembrolizumab in trials could be harder to achieve broadly, in I-MAT trial [36]. Clinicians should incorporate trial data flexibly, tailoring treatment plans to individual patient factors (age, immune status, comorbidities) and discussing the uncertainties of benefit magnitude in each context.

### 3.2. Recent Reviews, Guidelines and Major Prospective Clinical Trials

Patel et al. from New Orleans wrote an excellent overview of current MCC trials covering emerging strategies such as immunotherapy, intra-tumoral injections, altered radiation dosing and combination treatment [42]. The clinical trial landscape has shifted away from chemotherapy toward immune-based treatments. Front-line ICIs in trials show ~50–60% overall response rate (with ~20% complete response) and significantly improved 2-year survival (~60–70%) in advanced MCC. Most responders remain in remission at 1 year. However, about half of all patients still do not achieve durable control, so trials are exploring ways to extend benefits and overcome resistance, which will be discussed below. First-line ICI has replaced chemotherapy as the standard of care in MCC trials reflecting its superior efficacy. The review highlights active investigations into combination approaches-e.g., adding radiotherapy (RT), such as stereotactic body RT to ICIs or novel agents/injections—to boost responses and tackle primary or acquired resistance. Additionally, randomized trials (ADMEC-O, STAMP) are evaluating ICI in the adjuvant settings post-surgery to improve cure rates.

There are two very important and commonly used guidelines on MCC, from Europe and America, respectively. The European Society for Medical Oncology—European Reference Network for Rare Adult Solid Cancers [43] (*ESMO-EURACAN*) in partnership has detailed guidelines on MCC diagnosis, treatment, and follow-up. These focus on multidisciplinary care in specialized centers, given MCC’s aggressive nature and rising incidence in Europe. A comprehensive workup for MCC includes a full skin and lymph node exam, SLNB for clinically node-negative patients, and baseline fluorodeoxyglucose positron emission tomography/computeried tomography (FDG-PET/CT) imaging if available. CT of the chest, abdomen, pelvis ± head/neck MRI may be used for staging. Local therapy involves wide excision with 1–2 cm margins or Mohs surgery in select cases. Narrower margins (0.5–1 cm) may be acceptable if followed by adjuvant RT, which is recommended for all cases post-surgery to improve local control and survival.

SLNB should be performed before wide excision to preserve lymphatic mapping. If SLN is positive, completion lymph node dissection and/or nodal RT is advised after multidisciplinary discussion. Clinically involved nodes or MCC of unknown primary require dissection followed by RT, or definitive RT if inoperable. SLN-negative patients may be observed, but nodal RT can be considered if SLNB is not done or unreliable (e.g., head/neck primaries, immunosuppressed patients). Adjuvant chemotherapy is not recommended in stage I–III due to lack of survival benefit.

For unresectable stage III or metastatic stage IV MCC, immune checkpoint inhibitors (ICIs) are first-line. Avelumab, pembrolizumab, nivolumab, and retifanlimab have shown high response rates and durable remissions [44]. Avelumab and pembrolizumab are preferred in practice. If immunotherapy fails or is contraindicated, options include palliative RT, chemotherapy (e.g., platinum-etoposide), or best supportive care, ideally in a clinical trial. Surgery or stereotactic RT may be considered for oligometastatic disease.

ESMO reports 5-year overall survival (OS) rates of ~64% for stage I–II, ~51% for stage III, and 17–29% for stage IV. SLNB upstages ~30% of clinically node-negative patients and improves staging accuracy. Negative SLNB correlates with lower recurrence risk (~5–12%). Meta-analyses show surgery + RT reduces mortality (~19%) and improves locoregional control. RT benefits are strongest in early-stage disease.

Immunotherapy has transformed MCC outcomes. KEYNOTE-017 showed 58% overall response rate (ORR) with pembrolizumab and 3-year OS of ~59% [33]. JAVELIN Merkel 200 reported ~40% ORR with avelumab and median OS ~20 months [34]. Responses are often durable, while chemotherapy yields short-lived responses and significant toxicity.

Follow-up is crucial, especially in the first 3 years due to high relapse rates. ESMO recommends centralized care in high-volume centers, regular exams, and imaging for high-risk patients. Their evidence-based guidelines promote harmonized, multidisciplinary care to optimize MCC outcomes.

*The second guideline, National Comprehensive Caner Network NCCN* (v2.2025) is from the United States, developed by a multidisciplinary panel of expert from major cancer centers which includes Professor Paul Nyhiem who has published a lot of MCC research. Last updated on 18 April 2025, it reflects current evidence and FDA approvals. This summary emphasizes evidence-based, category 1/2A consensus recommendations for managing MCC in oncology practice, with a strong endorsement for clinical trial enrollment when appropriate. Adjuvant chemotherapy is not recommended for stage I–III MCC due to lack of survival benefit. For unresectable stage III or metastatic stage IV disease, ICI is the first-line treatment. Agents such as avelumab, pembrolizumab, nivolumab, and retifanlimab have shown high response rates and durable remissions. Avelumab and pembrolizumab are preferred in practice, although single-agent nivolumab is also useful based on the CheckMate 358 trial, a nonrandomied, open-label, phase I/II trial [45]. The 68 patients with recurrent/metastatic MCC were treated with nivolumab monotherapy (240 mg every 2 weeks). The results are ORR 60%, median DOR 60.6 months, median PFS 21.3 months, and median OS 80.7 months [45].

If immunotherapy fails or is contraindicated, options include palliative RT, chemotherapy (e.g., platinum-etoposide), or best supportive care—ideally within a clinical trial. Surgery or stereotactic RT may be considered for oligometastatic disease.

The Surveillance, Epidemiology, and End Results (SEER) data show five-year survival rates ranging from 76% (stage I) to 19% (stage IV) [46]. NCCN notes that initial PET/CT or CT imaging upstages 12–20% of patients, supporting routine imaging.

SLNB is positive in 30–40% of clinically node-negative cases. A negative SLNB correlates with lower recurrence risk and better survival. Adjuvant RT significantly reduces recurrence and improves outcomes, especially when margins are close. Fractionated RT may halve recurrence rates compared to single-dose RT, which corroborates studies from Tai et al. [47].

Follow-up every 3–6 months for the first three years is advised. Biomarkers such as MCPyV antibody titers and ctDNA may help detect recurrence earlier. NCCN guidelines emphasize multidisciplinary care, risk stratification, and personalized surveillance to optimize MCC management.

#### 3.2.1. Neoadjuvant Immunotherapy

The rationale for short-course preoperative immuno-oncology (IO) therapy is that it may induce significant tumor regression, allowing for less extensive surgical resection and improving long-term outcomes. Moreover, neoadjuvant therapy can prime the immune system by exposing it to intact tumor antigens, thereby enhancing systemic anti-tumor immunity. It also serves as an early in vivo test of therapeutic sensitivity for individual patients. If the pathological response is suboptimal, alternative adjuvant treatments can be selected postoperatively.

The *CheckMate 358* trial represents a landmark study in the neoadjuvant setting [13]. This phase I/II trial evaluated nivolumab, a PD-1 inhibitor, in patients with resectable MCC. Among 33 radiologically evaluable patients, 54.5% achieved tumor reductions exceeding 30%, with pathologic complete responses (pCR) observed in nearly half. In particular, neoadjuvant nivolumab was well tolerated, with no delays in planned surgery and minimal grade 3–4 adverse events.

#### 3.2.2. Adjuvant Immunotherapy (Table 2, Table 3 and Table 4)

The *ADMEC-O* phase II trial investigated adjuvant nivolumab versus observation in patients with completely resected MCC [18]. Conducted across 20 academic centers in Germany and the Netherlands, the study enrolled 179 patients, randomized in a 2:1 ratio to receive nivolumab or observation. Nivolumab or OPDIVO^®^ (480 mg) (Bristol Myers Squibb, New York, NY, USA) was administered intravenously every 4 weeks for up to 1 year (i.e., a maximum of 13 doses). At 12 months, disease-free survival (DFS) was 85% in the nivolumab group versus 77% in the control group. Although the difference did not reach statistical significance, the trend favored immunotherapy. *However*, 42% of patients receiving nivolumab experienced grade 3–4 adverse events, including rash and endocrine dysfunction. These findings emphasize the need for careful patient selection and monitoring, especially in the adjuvant setting where patients may be asymptomatic.

The *ADAM* phase III trial evaluates adjuvant avelumab, a PD-L1 inhibitor, in node-positive MCC patients following surgery and radiation [32]. This study, also known as the adjuvant MCC trial, is a multicenter, randomized, double-blinded, placebo-controlled trial involving patients with nodal metastases from MCC. Participants receive avelumab 10 mg/kg intravenously over 1 h every 15 days during Induction Phase 1 (days 0–120), every 30 days during Induction Phase 2 (days 121–240), and every 120 days during the Maintenance Phase, for up to a total of 720 days (approximately 2 years), provided there is no disease progression or unacceptable toxicity. The trial has completed accrual. Preliminary data suggest improved DFS, with common side effects including nausea, fatigue, and pruritus. The trial aims to clarify whether adjuvant IO can reduce recurrence risk in high-risk patients.

The Surgically Treated Adjuvant Merkel cell carcinoma with Pembrolizumab *(STAMP) trial* of the Dana-Farber Cancer Institute, another phase III study, has also completed accrual [35]. It assesses the use of pembrolizumab in patients with stage I–III MCC after complete resection (Table 2). It is one of the largest and most comprehensive efforts to define the role of adjuvant immunotherapy in early-stage MCC, potentially expanding its role beyond advanced MCC. The STAMP trial (NCT03712605) is led by U.S. institutions and coordinated by the ECOG-ACRIN Cancer Research Group [41], it includes a broad network of participating centers across the United States but does not list international sites.

**Table 2:** Summary of the Surgically Treated Adjuvant Merkel cell carcinoma with Pembrolizumab (STAMP) study (NCT03712605).

Study start: January 2019 Primary completion: completed accrual Study completion finish: 2026–2027 not publicly announced yet
Key Study Details
Primary Objective: Compare recurrence-free survival (RFS) and overall survival (OS) between pembrolizumab and standard observation. Design: phase III, randomized, placebo-controlled study Status: Closed to accrual; currently in follow-up phase Intervention: Arm A (Intervention): Pembrolizumab 200 mg intravenously every 21 days for up to 17 cycles (~1 year), with optional radiation therapy. Arm B (Control): Standard-of-care observation, with follow-up every 3 months for 1 year, then every 6 months for 5 years. Eligibility: Adults (≥18 years) with stage I–IIIb MCC. Must have undergone complete surgical resection within 16 weeks prior to randomization. Sentinel lymph node biopsy required for stage I patients. Accepts patients with unknown primary tumors if regional disease is present Secondary Objectives: Assess distant metastasis-free survival (DMFS), evaluate adverse events, analyze the impact of radiation therapy on outcomes Location and Sponsor: American centers. National Cancer Institute (NCI).

*The I-MAT* (NCT04291885), a randomized, placebo-controlled, phase II trial of adjuvant Avelumab in patients with stage I-III Merkel cell carcinoma aiming to explore the efficacy of avelumab as adjuvant immunotherapy to prevent recurrence (Table 3) [42]. Its comparison with STAMP trial is listed in Table 4 for clarity.

**Table 3:** Summary of I-MAT (NCT04291885) study in Merkel cell carcinoma.

Study start: 26 October 2020 Primary completion: 1 April 2027 Study completion finish: 1 April 2028
Key Study Details
Primary Goal: to evaluate whether avelumab, an anti–PD-L1 immunotherapy, can improve recurrence-free survival (RFS) following definitive local treatment. Design: Quadruple-masked, parallel assignment Intervention: Avelumab 800 mg IV every 2 weeks for 6 months vs. placebo Participants: 122 enrolled Eligibility: Adults (≥18 years) with histologically confirmed stage I–III MCC (clinical stage I; pathological stage I with positive lympho-vascular invasion (LVSI) only; clinical or pathological stage II and III. No distant metastases on PET/CT Primary Endpoint: RFS at 24 months Secondary Endpoints: Overall survival, disease-specific survival, loco-regional failure-free survival, distant metastasis-free survival, treatment toxicity, and patient-reported quality of life (FACT-M) Locations: Multiple sites in Australia and New Zealand, including major centers like the Peter MacCallum Cancer Centre and Royal Adelaide Hospital.

PET/CT, positron emission tomography/computerized tomography.

**Table 4:** Comparing the STAMP and I-MAT trials—two landmark studies evaluating adjuvant immunotherapy in early stages of Merkel cell carcinoma (MCC).

Feature	STAMP Trial (NCT03712605)	I-MAT Trial (NCT04291885)
Sponsor	ECOG-ACRIN/National Cancer Institute (USA)	Melanoma and Skin Cancer Trials (Australia/New Zealand)
Start Year	2019	2020
Status	Closed to accrual; in follow-up phase	Active, not recruiting
Participants	~280 patients	122 patients
Eligibility	Stage I–III MCC, completely resected	Stage I–III MCC
Intervention	Pembrolizumab 200 mg IV q21d × 17 cycles (~1 yr)	Avelumab 800 mg IV q2w × 6 mos
Control Arm	Standard-of-care observation	Placebo
Primary Endpoint	Recurrence-free and overall survival (RFS and OS)	RFS
Secondary Endpoints	DMFS, toxicity, QoL, impact of radiation	OS, DSS, toxicity, QoL
Geographic Scope	United States only	Australia and New Zealand
Radiation Therapy	Optional, per standard of care	Allowed, based on clinical indication
Follow-up Duration	5 years	2 years

DMFS, distant metastases free survival; DSS, disease-specific survival; ECOG-ACRIN, Eastern Cooperative Oncology Group—American College of Radiology Imaging Network; IV, intravenous; mos, months; OS, overall survival; q21d, every 21 days; q2w, every 2 weeks; QoL, quality of life; RFS, relapse-free survival; USA, United States of America.

#### 3.2.3. Primary Therapy and Salvage Use

As some patients with MCC are inoperable due to poor performance status or advanced disease stage, the use of immunotherapy has evolved over the years, accumulating sufficient clinical experience to support large-scale trials [48].

*The KEYNOTE-017 trial* evaluated pembrolizumab as first-line therapy in advanced MCC [33]. Among treatment-naïve patients, the ORR was 56%, with CR in 24%. At three years, PFS was approximately 40%, and OS exceeded 60%. These outcomes compare favorably to historical chemotherapy data, which typically yield short-lived responses and high toxicity. Pembrolizumab (KEYNOTE-017 Trial) is still investigational. While used as first-line, the trial also tracked salvage therapies post-progression. Patients who relapsed after pembrolizumab showed extended survival with subsequent immunotherapies or chemotherapy [33].

*The JAVELIN trial* assessed avelumab in patients with metastatic MCC who had progressed after chemotherapy [49]. The ORR was 33%, with durable responses in a subset of patients. Avelumab was well tolerated, with manageable immune-related adverse events. Importantly, responses were observed in both virus-positive and virus-negative tumors, suggesting broad applicability. The latest update was in 2024 [19]. Both *pembrolizumab* and *avelumab* have demonstrated efficacy as salvage therapies in patients who relapse after initial treatment [50]. Retreatment or switching agents may be considered, although data are limited. Standard strategies nowadays include chemotherapy, RT, surgery and combination immunotherapy.

*Dual ICI (ipilimumab + nivolumab)* has shown efficacy in the following studies: (1) ADOREG Registry Study. Ipilimumab plus nivolumab (ipi-nivo) in avelumab-refractory MCC: a multicenter study of the prospective skin cancer registry *ADOREG* evaluated 14 patients with advanced MCC who were resistant to avelumab. The combination of ipilimumab and nivolumab showed a 50% ORR, with durable responses and promising survival outcomes. (2) *CheckMate 358* Trial is a non-randomized, open-label, international, multicenter phase I/II study (13) which compared nivolumab alone vs. ipi-nivo in 68 patients. While response rates were similar (~60%), combination therapy had shorter duration of response and more side effects. The study suggests that combination therapy may not always improve outcomes. (3) The University of Pennsylvania Experience of use of combination ipilimumab/nivolumab for refractory Merkel cell carcinoma [51]. This single-institution experience is a retrospective analysis (with prospective elements) which reviewed 17 patients treated with ipi-nivo after progression on first-line therapy. The objective response rate was 36%, with manageable toxicity and some durable responses. (4) Combination with stereotactic RT, ipi-nivo demonstrated a 41% CR in previously treated patients in a landmark phase 2 study [52,53].

*Amrubicin* (Anthracycline-based Chemotherapy) is being investigated in platinum-refractory MCC [54]. A case report showed partial response and symptom improvement over 9 cycles, hence suggesting potential as a second line option for advanced MCC.

In summary, Table 5 below has important information for the clinicians at bed-side. During communication with intelligent patients, quoting some numbers will in-crease patient understanding, according to clinical experience of the authors.

### 3.3. Mechanisms of Resistance

Despite encouraging outcomes, immunotherapy resistance remains a significant challenge. Treatment resistance was defined by the Society of Immunotherapy for Cancer consensus recommendations [56]:

(1) *Primary resistance* (upfront progressive or stable disease with subsequent progression, having received at least 6 weeks and up to 6 months of anti-PD-(L)1 therapy).
(2) *Secondary resistance*—upfront partial or complete response with subsequent progressive disease, or stable disease for >6 months prior to progression disease, after at least 6 months of anti-PD-(L)1 therapy, with progression occurring ≤12 weeks of anti-PD-(L)1 therapy cessation.
(3) *Late progression*—if patient had upfront complete/partial response or stable disease for >6 months prior to progression, with progression occurring >12 weeks following anti-PD-(L)1 therapy discontinuation.

While not exhaustive, the following are several commonly proposed mechanisms of drug resistance:

(1) Intra-tumoral STING activation: While STING agonists can enhance immune responses, chronic activation may lead to immune exhaustion or paradoxical suppression [57]. The STING protein was found to be absent in MCC cells themselves, but present in the surrounding immune and stromal cells within the tumor microenvironment [58]. This suggests that STING activators may exert their effects indirectly in MCC, by signaling through these non-tumor cells, rather than acting directly on cancer cells [same as above*]. This observation also suggests that resistance may not be due to chronic activation within cancer cells, but rather to a direct lack of STING expression in the target cells, or that its effect is dose-dependent, as high doses can lead to reduced efficacy or even cause “adverse effects.” [59].
(2) Tumor-associated macrophages (TAMs) can create an immunosuppressive microenvironment, inhibiting T-cell infiltration and function [60], as noted by Professor Ann Silk, a leading expert in Merkel cell carcinoma (MCC).
(3) Loss of major histocompatibility complex (MHC) class I expression: Downregulation of antigen presentation impairs recognition by cytotoxic T cells [61,62].
(4) T-cell exhaustion: Chronic antigen exposure may lead to dysfunctional T cells, characterized by upregulation of inhibitory receptors (e.g., TIM-3, LAG-3) [63,64].
(5) Immunosuppressive cytokines: Elevated levels of IL-10 and TGF-β may dampen anti-tumor immunity [65,66].
(6) Understanding these pathways is critical for developing next-generation therapies, including combination checkpoint blockade, adoptive T-cell transfer and personalized vaccines [67,68].

### 3.4. What Measures Will Have the Greatest Impact on Improving Outcomes in MCC?

MCC shares clinical and biological characteristics with other aggressive skin cancers, notably melanoma. Immunotherapies such as nivolumab and pembrolizumab have demonstrated effectiveness across multiple stages of MCC presentation, leading to improved outcomes in some patients. Despite these advances, optimizing long-term prognosis still requires a multifaceted strategy.

Immunotherapy has significantly transformed the care of patients with MCC [69]. To date, our expert team proposes several actionable recommendations to improve MCC outcomes. First, increasing awareness among both healthcare professionals and the public is essential. MCC’s rarity and aggressive nature often delay recognition; timely education campaigns can promote earlier detection and intervention. Second, minimizing delays in diagnosis and ensuring swift referral to specialists is critical for initiating appropriate treatment before disease progression.

Third, stratifying patients into good- and poor-risk categories allows for personalized treatment planning based on tumor biology and clinical aggressiveness. Additionally, clinicians must carefully evaluate patients’ comorbid conditions to minimize both treatment-related toxicities and financial burdens—a concept now recognized as “financial toxicity.”

Regular follow-up is indispensable to monitor treatment response and detect recurrence. Emerging technologies such as circulating tumor DNA (ctDNA) assays, MCC polyomavirus viral titers, and advanced imaging modalities offer promising tools for early identification of disease relapse.

Cost-effectiveness must also guide the selection of diagnostic tests used for staging, surveillance, and re-staging at recurrence. Rational test utilization balances clinical benefit with resource stewardship. Finally, enhancing patients’ quality of life during and after treatment is vital. This includes minimizing adverse effects, supporting functional outcomes, and providing counseling that addresses psychological, sexual, and social well-being, as in the subsection below.

Taken together, this comprehensive approach aligns with a patient-centered paradigm and holds the greatest promise for improving both survival and quality of life in those affected by MCC.

### 3.5. Future Direction in Research

The future of MCC treatment should involve multimodal integration, with immunotherapy combined with other modalities to improve outcomes and to minimize toxicity. Here is how the landscape is evolving:

#### 3.5.1. Adoptive T-Cell Transfer

Adoptive T-cell transfer (ATT) represents a promising approach, particularly in cases of MCC associated with the Merkel polyomavirus (MPV). For example, a single-patient clinical trial demonstrated that combining HLA-I-enhancing agents with MHC-specific T-cell therapy resulted in tumor regression and delayed the onset of distant metastases [70]. However, challenges remain, such as low MHC class I expression on tumor cells, which can limit the efficacy of the transferred T cells [71]. Future trials aim to address these challenges to increase the effectiveness of this approach [72].

#### 3.5.2. Therapeutic Vaccines:

Therapeutic vaccines aim to stimulate a strong and specific immune response against cancer cells. They are a promising approach, especially in the context of resistance to current IO [73]. Various types of vaccines are being explored for MCC [74].

(1) Peptide-based vaccines: These vaccines consist of short peptide sequences of tumor antigens and require strong adjuvants to enhance the immune response. They are taken up by dendritic cells, which present them to T cells.
(2) mRNA vaccines: These vaccines use synthetic mRNA at the desired antigen concentration and are typically in a lipid-based compound. They have a good safety profile and the ability to rapidly stimulate the immune system.
(3) Vaccines based on oncolytic viruses: These vaccines aim to directly infect and destroy cancer cells, leading to the release of tumor antigens and stimulating an immune response. Examples include modified herpes simplex virus (RP1) [75,76] and oncolytic adenovirus (MEM-288) [77].
(4) Plasmid/viral vector vaccines: These use a virus to deliver genetic material that elicits an antigen, leading to an immune response. They can improve targeting of treatments to tumor sites and avoid excessive immune activation.
(5) Exosome-based vaccines: These use extracellular vesicles carrying membrane proteins to increase the immune response and can stimulate T cells similarly to dendritic cells.

Many of these vaccines are being combined with ICIs in ongoing studies to enhance immune responses and counteract resistance. However, enhancing immune responses through combination therapy may increase the risk of serious adverse events, such as those seen with nivolumab and ipilimumab (such as immune hypophysitis, thyroiditis, colitis, and hepatitis). Cytokine release syndrome is also a possibility [78,79]. However, directing the immune response specifically to cancer cells via vaccination may reduce some of the toxicities of ICIs.

#### 3.5.3. Combining Different Treatment Modalities

*RT* has a synergistic effect as it may increase tumor antigen presentation, making cancer cells more recognizable to the immune system [80]. In addition, shrinkage of the tumor will allow systemic therapeutic agents to reach cancer cells. By using different modalities can also decrease side effects if individual component of the combined therapy can be reduced in intensity, in terms of drug/RT dose or RT treatment volume. Careful design of clinical trials is needed as toxicities could increase with combined treatment. Current efforts such as the NCT03304639 study which is testing pembrolizumab with stereotactic body radiation therapy (SBRT) to improve PFS [81].

*Adjuvant RT* may consolidate local control while immunotherapy targets systemic disease. Research should also focus on selecting the proper patients for postoperative RT with careful considerations on dose and volume.

Regarding chemo-immunotherapy combinations, many experts express concern about the immunosuppressive effects of chemotherapy and the diminished responses to immuno-oncology agents following prior chemotherapy. However, chemotherapy can reduce tumor burden and may act synergistically with immunotherapy by enhancing cytotoxic effects. Ongoing trials include the MERCURY (NCT05594290) evaluating retifanlimab with cisplatin and etoposide before surgery [82].

*Lutetium-177 dotatate* (a radiolabeled peptide) is tested in combination with avelumab or pembrolizumab in trials such as GoTHAM and iPRRT [83,84]. The combined treatment may confer targeted cytotoxicity while activating immune responses.

#### 3.5.4. Biomarkers

Viral titers and ctDNA are used to monitor recurrence and guide treatment intensity in some well-off countries, such as the United States [85]. Recent data suggest that baseline levels of ctDNA may serve as early predictors of immunotherapy response, while longitudinal monitoring enables real-time assessment of minimal residual disease and recurrence. In a recent study, ctDNA positivity preceded radiographic relapse by several weeks, supporting its role as a dynamic biomarker in MCC surveillance [86].

PD-L1 status and MCPyV may help predict response to checkpoint inhibitors; however, access remains limited—even in Canada, where a significant proportion of the national budget is allocated to healthcare. There are long waiting lists for new consultations with medical oncologists, and subsequent biomarker testing on tissue specimens often involves turnaround times of several weeks. Although reflex ordering has been discussed, it has yet to be implemented in many smaller or rural hospitals across Canada.

#### 3.5.5. Improving Quality of Life During and After Treatment

The following reflects expert opinions on minimizing complications and improving quality of life for patients with MCC. Infusion reactions to immunotherapy or chemotherapy are relatively common, and pre-medication protocols could be optimized through further research.

Rehabilitation is crucial to address both physical and psychosexual impacts of aggressive treatments like surgery and radiation. Maxillofacial prostheses (e.g., nose, ear) may be used following facial resection to restore aesthetic form and function, significantly boosting patient confidence. Lymphedema can be prevented through lymphovenous anastomosis and managed with physiotherapy, including exercise with or without compression garments. Physiotherapy also plays a critical role in addressing facial nerve damage and improving facial symmetry and expression.

Rehabilitation extends to psychological well-being, addressing body image concerns, anxiety, and relationship distress through psychosexual counseling and behavioral therapies [87,88]. Pelvic RT frequently results in long-term sexual dysfunction. Women may experience vaginal dryness, pain, and stenosis, while men may develop erectile dysfunction or reduced libido. Pelvic floor physiotherapy can be transformative for painful intercourse and overall pelvic health. Other effective rehabilitative strategies for female patients include vaginal lubricants, estrogen creams, and vaginal dilators to prevent vaginal obliteration or stenosis. Oncologists should provide these dilators and reinforce their use during follow-up visits. Successful implementation requires a multidisciplinary approach involving nurses and social workers as well.

For men, treatments for erectile issues are available. To improve post-treatment health, various modern rehabilitations are available. For example, erectile impotence can be treated by oral phosphodiesterase type-5 inhibitors as first-line treatment [89] or Eroxon gel [90]. The latter is available over the counter and contains ethanol, propylene glycol and glycerine/glycerol. It works within 10 min, with few side effects apart from local skin reactions [91,92]. Other treatments are lifestyle interventions, psychological counseling and Kegel exercises. More invasive treatments include self-injections and penile implants (a surgical procedure available in most large Canadian cities).

Last but not the least, since patients with MCC often experience recurrence and poor outcomes, effective communication skills among healthcare workers are essential [93,94]. Ongoing medical and nursing education remains critically important [95,96]. Caring for cancer patients is both an art and a science.

In summary, a proactive multidisciplinary approach and open communication among healthcare team members and the patients are essential for optimizing patients’ quality of life following treatment. The examples presented above relate to situations in the Middle East, the United States, and Canada—regions in which members of this research team received training. It is our hope that these practices can be applied globally, with broad generalizability.

## 4. Conclusions

This comprehensive review provides useful bedside and research information, meant to help both healthcare workers and patients. Immunotherapy has significantly advanced the management of MCC across neoadjuvant, adjuvant, and advanced settings.

Neoadjuvant setting: Immune checkpoint inhibitors like nivolumab have shown high response and pathologic complete response rates without delaying surgery. This approach is especially promising for bulky or node-positive disease and should be pursued in experienced centers or clinical trials with coordinated multidisciplinary care.

Adjuvant setting: For resected high-risk MCC, adjuvant immunotherapy may reduce recurrence, as early trials (e.g., ADMEC-O) suggest improved disease-free survival. However, immune-related toxicities are common, making careful patient selection and monitoring essential.

Advanced disease: PD-1/PD-L1 inhibitors are now first-line therapy for advanced MCC, offering durable responses (~60% ORR) and prolonged survival compared to historical chemotherapy.

Special populations: Immunosuppressed and autoimmune patients may benefit from immunotherapy but face higher risks. Individualized assessment and specialist input are critical.

Multimodal integration: Immunotherapy should complement, not replace, surgery and RT. Neoadjuvant, adjuvant, and palliative combinations are feasible and potentially synergistic, requiring multidisciplinary coordination for optimal outcomes. Chemotherapy may still have a role in MCC: Ongoing trials include the MERCURY (NCT05594290) evaluating retifanlimab with cisplatin and etoposide before surgery, and the PANDORA trials is also testing synergistic effects of chemotherapy with ICI on MCC. *Amrubicin* (Anthracycline-based chemotherapy) is also being investigated.

## Figures and Tables

**Table 1:** Summary of immune checkpoint inhibitor studies in Merkel cell carcinoma.

References	Setting	Intervention	Outcome/Results	Keypoints
**Neoadjuvant**				
Topalian S.L. (2020) [13]	Neoadjuvant	Nivolumab	50–60% pCR in resectable MCC. Significant tumor downsizing	Neoadjuvant IO can achieve high rates of pCR, potentially simplify surgery and improve outcomes in localized disease
Bhatia S. (2020) [14]	Neoadjuvant (cohort A)	Intratumoral 1L-12 plasmids DNA via electroporation (tavo-EP)	Objective response in injected and non-injected tumors. Demonstrates initial safety/efficacy in early-stage MCC	Intratumoral IO is a promising approach for inducing local and systemic anti-tumor responses, relevant for neoadjuvant strategies
Kuchimanchi N. (2025) [15]	Neoadjuvant	Preoperative nivolumab induced rare dysautonomia treated with IVIG	57-year-old patient developed dysautonomia (orthostatic hypotension, urinary retention, hearing loss, diplopia) after 2 cycles. IVIG markedly improved neurological symptoms. It highlights both the efficacy of neoadjuvant ICI and the need to manage uncommon severe side effects.	A very rare toxicity of ICI CC. Importance of vigilant monitoring for irAEs and rapid intervention. Early IVIG treatment in this case reversed the dysautonomia, suggesting that timely diagnosis and treatment of irAEs can allow patients to continue benefiting from ICI without lasting harm.
Correa Roa C. (2025) [16]	Neoadjuvant for loco-regional recurrence	Preoperative nivolumab for 6 cycles	80-year-old patient with arm lesion and axillary nodes. Surgery found only scar tissue: pCR in tumor bed or nodes, and no further recurrence after resection	High immunologic sensitivity of MCC: even aggressive recurrence achieves pCR; “revolutionizing” treatment for advanced and recurrent MCC.
**Adjuvant**				
Becker J.C. (2023) [17,18]	Adjuvant (ADMEC-O)	Nivolumab vs. observation	Improved DFS after complete resection of MCC, reduces recurrence. OS results not mature yet	Significantly improves DFS in resected MCC, establishing a new SOC for high-risk patients. Support further adjuvant trials.
**Primary and salvage therapy in advMCC:** see the text for more details.				
D’Angelo S.P. (2021) [19]	Primary/salvage	Avelumab	Updated OS data > 5 years: durable responses, with many long-term responders	Avelumab: long term survival benefits in mMCC, including previously treated patients
Nghiem P.T. (2016) [10]	Primary/salvage	Pembrolizumab	56% ORR with durable responses. First evidence for PD-1 blockade in advMCC	Pembrolizumab is highly effective in advMCC, as new treatment option
Gaiser M.R. (2018) [20]	Primary/salvage	Avelumab (review)	~33% ORR in refractory disease, <60% in treatment-naïve cases; avelumab is safe	Avelumab is cornerstone of metastatic MCC treatment; effective in both 1L and refractory settings
D’Angelo S.P. (2021) [21]	Primary	Avelumab (1L)	*4-year FU*: sustained responses and long-term OS in 1L treatment of mMCC	Avelumab as 1L therapy: durable responses and prolonged survival
Kaufman H.L. (2016, 2018) [22,23]	Salvage	Avelumab (previously treated)	Updated efficacy results after ≥1 year FU up confirmed durable responses in chemo-refractory patients	Avelumab offers durable responses for progression after prior chemotherapy
Shirley M. (2018) [24]	Primary/salvage	Avelumab	Avelumab approval, efficacy and safety profile in metMCC	Reaffirms its role as the first approved IO drug for MCC, effective across treatment lines
D’Angelo S.P. (2020) [25]	Salvage	Avelumab (previously treated)	Long-term data and biomarker analyses: durable responses and insights into response predictors	Data supports avelumab benefits, with potential for biomarker-guided therapy in salvage settings
D’Angelo S.P. (2018) [26]	Primary	Avelumab (1L)	Interim analysis: ~62% ORR and manageable safety profile in 1L setting.	Avelumab is an effective and safe 1L option for metMCC
Nghiem P. (2019) [27]	Primary	Pembrolizumab (1L)	Durable tumor regression, improved OS as 1L therapy in advMCC	Pembrolizumab offers durable benefits as 1L treatment for adv MCC.
D’Angelo S.P. (2021) [26]	Primary ICI metastatic disease	Avelumab (1L)	39 patients, primary and biomarker analyses of 1L avelumab, showing high ORR/DOR	Detailed insight into 1L avelumab efficacy and potential biomarkers for responses
D’Angelo S.P. (2025) [28]	Salvage (progress post IO)	Management strategies post-PD-L1 progression	Discuss clinical outcomes and management for disease progression after initial IO	Crucial for understanding next steps and “salvage use” after primary IO failure
Mo J. (2025) [28]	Same as above	Same as above	Same as above	Same as above
**General/contextual reviews:** Topalian S.L. (2012 [29], 2015 [30]) were excluded after critical appraisal since they are not specifically for MCC.				
Lebbé C. et al. (2015) [31]	General MCC treatment	European consensus guideline	Covers diagnosis, treatment, and evolving role of systemic therapy	Includes the integration of IO into overall treatment regimen
Aquino de Moraes F. (2024) [9]	Same as above	ICI systemic review and metaanalysis	Efficacy and safety of ICI	Confirms the overall efficacy and safety of ICI

1L: first line, 2L: second line, advMCC: advanced MCC, DFS: disease-free survival, DNA: deoxyribonucleic acid, DOR, duration of response, FU: follow-up, ICI: immune checkpoint inhibitor, IO: immune-oncology, IVIG: intravenous immunoglobulin, MCC: Merkel cell carcinoma, metMCC: metastatic MCC, ORR: objective response rate, OS: overall survival, pCR: pathological complete response, PD-1: programmed cell death protein 1, PD-L1: programmed death-Ligand 1, SOC: standard of care, EP: electroporation.

**Table 5:** Comparing latest clinical trials for advanced MCC (advMCC).

References	Settings	Intervention	Outcome/Results	Key Points
Nyhiem P. (2021) [50]	Multicenter phase II trial (CITN-09/KEYNOTE-017), US; 50 patients with advanced unresectable/metastatic MCC. Median follow-up 31.8 months.	First line pembrolizumab (2 mg/kg IV every 3 weeks, up to 2 years)	ORR: 58% (30% CR, 28% PR). Median PFS: 16.8 months, 3-years PFS: 39.1%, 3-years OS: 59.4% overall; 89.5% in responders. Median OS not reached. Salvage therapies (chemo/ICI) extended survival in some resistant/relapsed patients.	Longest follow-up for first-line PD-1 blockade. High/durable response rates; most responses persisted ≥3 years. ECOG 0, greater tumor shrinkage, completion of 2 years, and low NLR linked to better survival. Pembrolizumab markedly outperforms historical chemotherapy outcomes.
Oldani S. (2025) [55]	Italy (multicenter- open-label phase II PANDORA trial led by National Cancer Institute, Milan) (NCT06086288)	First line combination chemo-immunotherapy (pembrolizumab platinum [cisplatin/carboplatin] +etoposide) in advanced MCC	In progress, evaluating whether *adding chemotherapy* to upfront immunotherapy improves outcomes in metastatic MCC. Primary endpoint is objective response rate; secondary endpoint includes OS, PFS, DOR. Plan to enroll 35 patients. Rationale: a significant subset of patients develop resistance to single-agent PD-1/PD-L1 therapy, so combining it with chemo (for its quick tumor shrinkage effect) may yield higher response rates and longer control.	Addresses a key unmet need in MCC treatment: improving first line efficacy for those not responding to IO alone. By leveraging the potential synergy of chemo-immunotherapy, PANDAORA aims to enhancing initial tumor response and possibly survival, compared to either modality alone. If successful, this combo approach could redefine the therapeutic standard for advMCC, offering new hopes for patients who historically had limited options.
Grignani G. (2025) [45]	Multiregional, open label single-arm phase II trial; 34 sites across USA, Canada, and Europe. Patients ≥ 18 y with recurrent locally advanced or metastatic MCC, chemotherapy—naïve, ECOG 0–1.	Retifanlimab 500 mg IV every 4 weeks (Q4W), for up to 2 years.	101 patients enrolled (2019–2021). ORR 54.5% (CR 17.8%, PR 36.6%); DCR 60.4%. median DOR: not reached (CR)/25.3 months (PR). Median PFS: 16.0 months; median OS: not reached, 63% alive at 3 years. Safety: grade ≥ 3 immune-related AEs in 10.9%.	Retifanlimab produced frequent, durable responses in advanced MCC, with survival benefit and manageable safety. Represents a new PD-1 inhibitor option for chemotherapy—naïve MCC patients.

1L: first line, 2L: second line, advMCC: advanced MCC, AEs: adverse events, DFS: disease-free survival, DNA: deoxyribonucleic acid, DOR, duration of response, ECOG: Eastern Cooperative Oncology Group, FU: follow-up, ICI: immune checkpoint inhibitor, IO: immune-oncology, IVIG: intravenous immunoglobulin, MCC: Merkel cell carcinoma, metMCC: metastatic MCC, ORR: objective response rate, OS: overall survival, pCR: pathological complete response, PD-1: programmed cell death protein 1, PD-L1: programmed death-Ligand 1, PFS, progression-free survival, PR: partial response, SOC: standard of care, USA: United States of America.

## Supplementary Materials

The following supporting information can be downloaded at: https://www.mdpi.com/article/10.3390/cancers17193272/s1, Table S1. Critical Appraisal of Reviewed Studies. Highlighted two Topalian studies not specifically for Merkel cell carcinoma. The Nghiem study will be explained in greater detail in the text of the paper.

## Abbreviations

The following abbreviations are used in this manuscript:

1L	First-line therapy
2L	Second-line therapy
ADMEC-O	Adjuvant Merkel Cell Carcinoma Nivolumab Trial
ADAM	Adjuvant Avelumab Merkel Cell Carcinoma Trial
ATT	Adoptive T-cell Transfer
ctDNA	Circulating Tumor DNA
DFS	Disease-Free Survival
DMFS	Distant Metastasis-Free Survival
DNA	Deoxyribonucleic Acid
DOR	Duration of Response
DSS	Disease-Specific Survival
ECOG	Eastern Cooperative Oncology Group
EP	Electroporation
FDA	Food and Drug Administration of the United States
ICI	Immune Checkpoint Inhibitor
IO	Immune-Oncology
I-MAT	Immunotherapy Merkel Adjuvant Trial
JAVELIN	JAVELIN Merkel 200 Avelumab Trial
KEYNOTE-017	Pembrolizumab Phase II Trial in Advanced MCC
LVSI	Lymphovascular Space Invasion
MCC	Merkel Cell Carcinoma
MCPyV	Merkel Cell Polyomavirus
metMCC	Metastatic Merkel Cell Carcinoma
mRNA	Messenger Ribonucleic Acid
NOS	Not otherwise specified
ORR	Overall Response Rate
OS	Overall Survival
pCR	Pathological Complete Response
PD-1	Programmed Cell Death Protein 1
PD-L1	Programmed Death-Ligand 1
PFS	Progression-Free Survival
PRISMA	Preferred Reporting Items for Systematic Reviews and Meta-Analyses
q21d	Every 21 Days
q2w	Every 2 Weeks
QoL	Quality of Life
RFS	Recurrence-Free Survival
SBRT	Stereotactic Body Radiation Therapy
SOC	Standard of Care
STAMP	Surgically Treated Adjuvant Merkel Cell Carcinoma with Pembrolizumab Trial
STING	Stimulator of Interferon Genes
TAM	Tumor-Associated Macrophages
TGF-β	Transforming Growth Factor Beta
UV	Ultraviolet Radiation
WHO	World Health Organization
