# Supplementary material for: Immune Checkpoint Inhibitors in Merkel Cell Carcinoma of the Skin: A 2025 Comprehensive Review
Source: Cancers (Basel). 2025 Oct 9;17(19):3272. doi: 10.3390/cancers17193272 (PMC12523286; doi:10.3390/cancers17193272)
Supplement: Supplementary file 1 [file cancers-17-03272-s001.zip › cancers-3791150-supplementary.pdf]

Supplementary Table S1. Critical Appraisal of Reviewed Studies. Highlighted two Topalian studies not specifically for Merkel cell carcinoma. The Nghiem study will be explained in greater detail in the text of the paper.

(JBI) Critical Appraisal Checklist for Included													
**Study**	**Type**	**Q1**	**Q2**	**Q3**	**Q4**	**Q5**	**Q6**	**Q7**	**Q8**	**Q9**	**Q10**	**Q11**	**Total %**	**Overall appraisal (√: included X: excluded)**
D'Angelo (2018)	Phase II trial (single arm)	Y	NA	Y	N	Y	Un	Y	Y	Y			75%	√
Nghiem (2019)	Phase II trial (single arm)	Y	NA	Y	N	Y	Y	Y	Y	Y			88%	√
D'Angelo (2020)	Phase II trial (single arm)	Y	NA	Y	N	Y	Y	Y	Y	Y			88%	√
D'Angelo (2021)	Phase II trial (single arm)	Y	NA	Y	N	Y	Y	Y	Y	Y			88%	√
Oldani (2025)	Phase II trial protocol	Y	NA	Y	N	Y	Y	Y	Y	Y			88%	√
Correa Roa (2025)	Case report	Y	Y	Y	Y	Y	Y	N	Y	NA			88%	√
Lugowska (2024)	Clinical guidelines (ESMO)	Y	Y	Y	Y	Y	Y	NA	NA	NA			88%	√
NCCN (2025)	Clinical guideline	Y	Y	Y	Y	Y	Y	NA	NA	NA			100%	√
Shirly (2018)	Drug review (narrative)	Y	Y	Y	Y	Y	Y	NA	NA	NA			100%	√
Colungo (2018)	Narrative review	Y	Y	Y	Y	Y	Y	NA	NA	NA			100%	√
Topalian (2020)	Phase I/II (single arm)	Y	NA	NA	NA	Y	Y	NA	Y	Y			100%	√
D'Angelo (2021)	Phase II Trial (single arm)	Y	NA	NA	NA	Y	Y	NA	Y	Y			100%	√
De Moraes (2024)	Systematic review & metal analysis	Y	Y	Y	Y	Y	Y	Y	Y	Y	Y	Y	100%	√
Colunga (2018)	Narrative review	Y	Y	Y	Y	Y	NA						100%	√

Nghiem (2016)	Phase II trial (single-arm)	Y	NA	NA	NA	Y	Y	NA	Y	Y			100%	√
Topalian (2012)	Phase I trial (dose-escalation)	Y	NA	NA	NA	Y	NA	NA	Y	Y			80%	X
Topalian (2025)	Narrative review	Y	Y	Y	Y	Y	NA						100%	X
Gaiser (2018)	Narrative review	Y	Y	Y	Y	Y	NA						100%	√
Lebbe (2015)	Clinical practice guidelines	Y	Y	Y	Y	Y	Y	Y					100%	√
D'Angelo (2024)	Phase II trial (single arm follow up)	Y	NA	NA	NA	Y	Y	NA	Y	Y			100%	√
Bahtia (2020)	Piolt clinical trial (phase I ,single arm)	Y	Y	Y	N	Y	Y	Y	Y	Y			89%	√
Kaufman (2018)	Phase II clinical trial (single arm	Y	Y	Y	N	Y	Y	Y	Y	Y			89%	√
Patel (2025)	Narrative review /expert opioin	Y	Y	Y	Y	Y	NA						83%	√
Kuchimanchi (2025)	Case report	Y	Y	Y	Y	Y	Y	Y	Y				100%	√
Nghiem (2021)	Phase II Clinical trial	Y	Y	Y	Y	Y	Y	Y	Y	Y			100%	√

Abbreviations: JBI: Joanna Briggs Institute, N: No, NA: Not available, Y: Yes.
